# Enzymatic degradation of biopolymers in amorphous and molten states: mechanisms and applications

**DOI:** 10.1002/2211-5463.70177

**Published:** 2025-12-10

**Authors:** Anđela Pustak, Aleksandra Maršavelski

**Affiliations:** ^1^ Radiation Chemistry and Dosimetry Laboratory, Division of Materials Chemistry Ruđer Bošković Institute Zagreb Croatia; ^2^ Department of Chemistry, Faculty of Science University of Zagreb Croatia

**Keywords:** amorphous state, biopolymers, enzyme‐catalyzed degradation, molten state, polymer crystallinity, thermal transitions

## Abstract

Plastic waste from fossil‐derived polymers remains a major environmental challenge, driving interest in biopolymers and enzyme‐enabled end‐of‐life strategies. This review synthesizes current understanding of how polymer structure and thermal state govern enzymatic degradability, with emphasis on semicrystalline architectures and state‐dependent accessibility. Within the Keller–Flory two‐phase framework, crystalline lamellae embedded in an amorphous matrix dictate water/enzyme diffusion, chain mobility, and hydrolysis kinetics. Enzymatic attack preferentially initiates in amorphous regions, producing characteristic biphasic behavior as amorphous domains erode faster than crystalline regions, leading to crystallinity enrichment and subsequent slowing of degradation. Thermal transitions further modulate this balance: near or above *T*_g_, segmental mobility and free volume rise, accelerating hydrolysis if enzymes remain stable; above *T*_m_, chain mobility is maximal, but enzyme stability typically limits feasibility. Processing and architecture also strongly influence outcomes: annealing increases crystallinity and slows mass loss, quenching suppresses crystallization and hastens degradation, random copolymerization disrupts packing and lowers *T*_m_, while block copolymers often degrade selectively by domain. Recent advances expand the operational window toward rubbery or near‐molten states for low‐melting aliphatic polyesters (e.g., PCL, PLGA, PEG‐*b*‐PLA), leveraging thermophilic/engineered hydrolases (cutinases, PETases, lipases, carboxylesterases) with demonstrated stability at 60–90 °C. Emerging strategies—including enzyme thermostabilization, AI‐guided design, disulfide grafting, smart encapsulation, and *in‐situ* enzyme embedding—enable self‐degradation of materials and accelerate inside‐out depolymerization under mild triggers. Integrating thermal analysis with polymer morphology and enzyme engineering offers a path to programmable, circular end‐of‐life for biopolymers, translating fundamental structure–property–reactivity relationships into practical enzymatic recycling and reduced environmental impact.

## Graphical abstract

This review explains how polymer morphology and thermal state shape enzymatic degradation pathways, comparing amorphous and molten biopolymer structures. By integrating structure–reactivity principles with insights from thermodynamics and enzyme engineering, it highlights mechanisms that enable efficient polymer breakdown. These concepts support the development of recyclable bioplastics and advance progress toward sustainable, circular polymer economies.

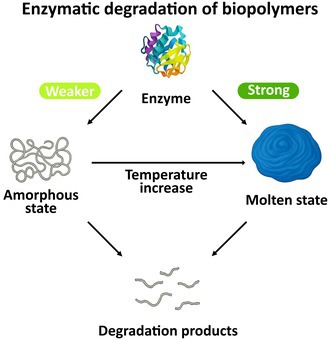

**Abbreviations**
CA: cellulose acetate
DMA: dynamic mechanical analysis
DSC: differential scanning calorimetry
DTA: differential thermal analysis
IR: infrared spectroscopy
LCC: leaf‐branch compost cutinase
MW: molecular weight
NMR: nuclear magnetic resonance
PCL: poly(caprolactone)
PCL‐co‐PLA: poly(caprolactone)‐co‐poly(lactic acid)
PE: polyethylene
PEG‐b‐PLA: poly(ethylene glycol)‐*b*‐poly(lactic acid)
PET: polyethylene terephthalate
PGA: poly(glycolic acid)
PHA: poly(hydroxyalkanoate)
PHB/PHBV: poly(hydroxybutyrate) (valerate)
PLA: poly(lactic acid)
PLGA: poly(L‐lactide‐*co*‐glycolide)
PLLA: poly(L‐lactic acid)
PP: polypropylene
*T*_c_
: crystallization temperature
*T*_g_
: glass transition temperature
TGA: thermogravimetric analysis
*T*_m_
: melting temperature
TPS: starch thermoplastic
*x*_c_
: degree of crystallinity
XRD: X‐ray diffraction

Plastic pollution from fossil fuel–derived materials has become a pressing environmental and public health issue, driving urgent demand for ecological and sustainable alternatives. In this context, biopolymers—materials derived from renewable natural sources—have emerged as promising substitutes for conventional fossil‐based polyolefin plastics. Often referred to as bioplastics, bio‐based polymers encompass a wide range of materials that are generally categorized into two groups: bio‐based but non‐biodegradable (43.7% of global bioplastics production in 2024) and bio‐based and biodegradable (56.3%) (Fig. 1).

**Fig. 1:**
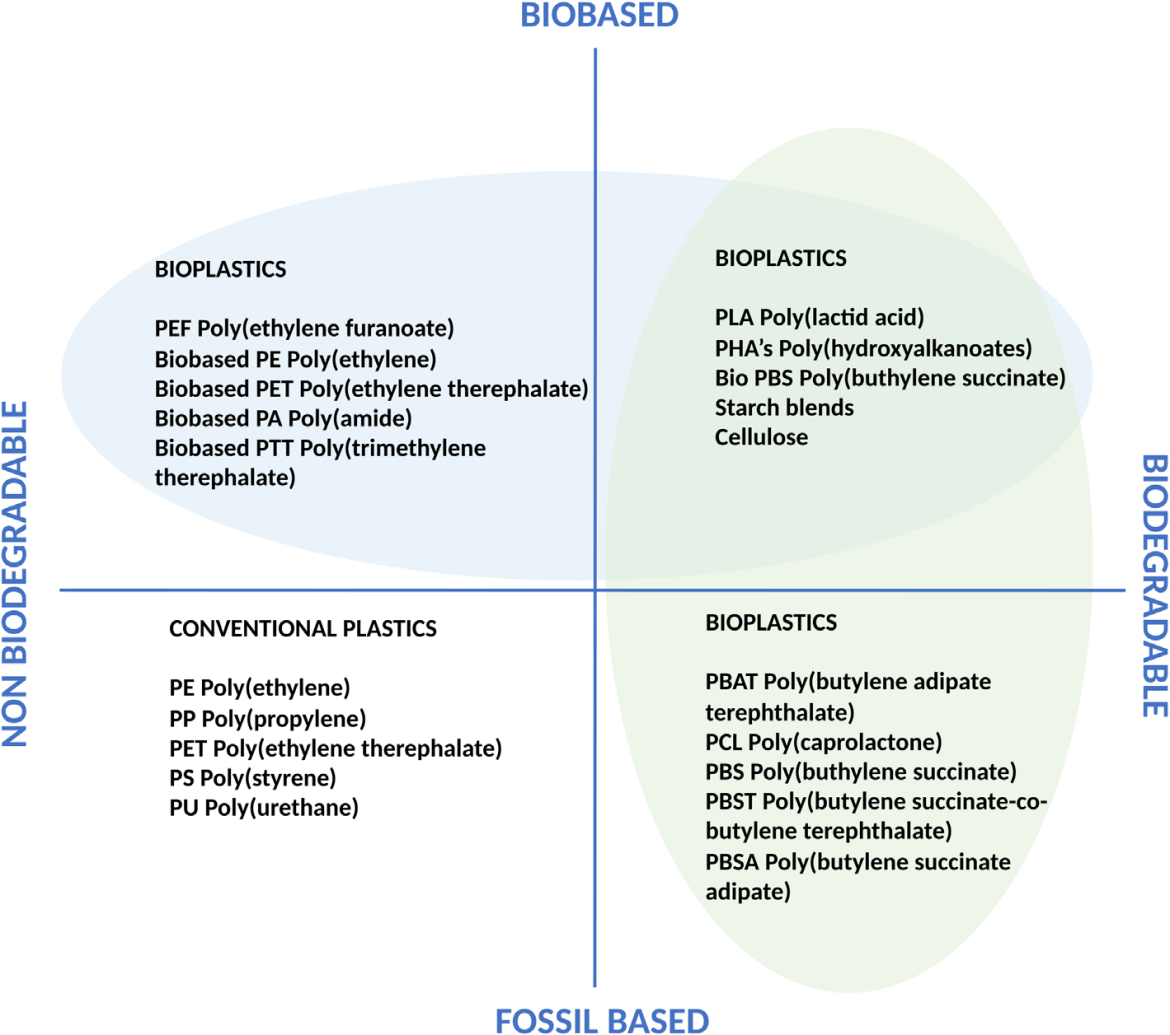
Plastic materials classification concerning origin and biodegradability (adapted from European Bioplastics) [1].

Global bioplastics production in 2024 is estimated at 2.5 million tonnes [1]. According to IUPAC, a biodegradable polymer is “a polymer susceptible to degradation by biological activity, with the degradation accompanied by a lowering of its molar mass” [1, 2]. In practice, international legislation defines biodegradability through performance‐based standards that specify: (a) required end products of degradation (e.g. CO_2_, water, biomass, and under anaerobic conditions methane); (b) timeframes for mineralization; (c) environmental conditions—industrial composting, home composting, soil, marine, or landfill—under which degradation must be demonstrated and (d) ecotoxicity and residue safety [3]. It is also important to distinguish compostability from biodegradability: while biodegradability refers solely to the material's capacity to mineralize through biological processes, compostability requires additional testing for physical disintegration and verification that no ecotoxic effects arise from the resulting compost.

Biopolymers—produced either by living organisms or synthesized from renewable feedstocks—are structurally and functionally diverse. They are commonly classified according to monomer chemistry, biological origin, production method, and thermal or mechanical properties [4, 5]. Although the production of bioplastics is steadily increasing, matching the mechanical, thermal, rheological, and other functional properties of conventional polyolefins such as poly(ethylene) (PE), poly(propylene) (PP), and poly(ethylene terephthalate) (PET) remains a major challenge [6, 7]. As a result, biopolymers are gaining market share primarily in single‐use packaging applications, while traditional polyolefins continue to dominate in high‐performance sectors like automotive, construction, and furniture manufacturing [1].

The ever‐increasing use of biopolymers highlights the urgent need to investigate degradation mechanisms—particularly microbial and enzymatic pathways—that can support sustainable waste management and enable the transition toward a circular economy.

## Polymer structure and phase states: crystalline vs. amorphous

**Fig. 2:**
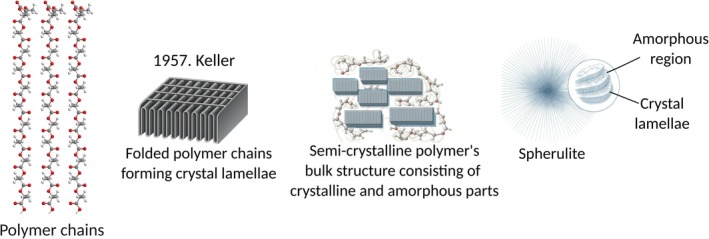
Scheme of two‐phase model describing the polymer structure. Polymer chains are folded in 10 nm thick layers called crystal lamellae that continue to grow as crystalline components. Between crystal regions, polymer chains remain unorganized, forming the amorphous component of the polymers' structure. These lamellae stack together to form crystallites – small, ordered domains where polymer chains are tightly packed in a regularly repeating structure – while the amorphous areas between lamellae, by contrast, represent disordered, non‐crystalline areas [76]. The two‐phase model provides the structural foundation for understanding the formation of higher‐order supramolecular morphologies during polymer crystallization. Upon cooling from the melt or precipitation from a dilute solution, crystalline and amorphous regions further self‐organize into complex structures such as spherulites, dendrites, or axialites [77, 78, 79, 80].

The supermolecular architecture of semicrystalline polymers is most often explained by the *two‐phase model* introduced by Keller and Flory, which describes the polymer as a system of discrete crystalline lamellae embedded within an amorphous matrix [8, 9]. Each lamella consists of polymer chains that fold back and forth in a regular, three‐dimensional arrangement, while the surrounding interlamellar amorphous regions contain randomly coiled and entangled chains lacking long‐range order (Fig. 2).

Although the two‐phase model (Fig. 2) simplifies polymer structure by considering only crystalline and amorphous phases without explicitly considering interfacial regions, it effectively captures the structural dichotomy that determines many materials' macroscopic properties—including tensile strength, barrier performance, thermal behavior, and biodegradability. The physical behavior of a polymer is thus closely tied to the relative proportions and spatial distribution of these two domains [10, 11, 12].

### Hierarchical organization and enzymatic degradation implications

The hierarchical structure of semicrystalline polymers presents substantial obstacles to enzymatic attack. Crystalline lamellae act as physical barriers that strongly restrict water and enzyme diffusion, thereby slowing or even preventing biodegradation. The densely packed crystalline cores of spherulites are particularly resistant to enzymatic attack, acting as protective shields that restrict enzyme penetration into the bulk material [6, 13, 14]. This structural organization amplifies the barrier effect by enclosing amorphous zones within crystalline networks, thereby limiting access to hydrolysable bonds. Consequently, enzymatic degradation is typically initiated in the more accessible amorphous regions—such as interlamellar spaces or interspherulitic boundaries—provided that enzymes can penetrate through the surrounding crystalline network. This preferential attack pattern underscores the critical importance of polymer morphology in determining biodegradation rates and pathways [13, 14, 15, 16].

The crystallinity‐to‐amorphous ratio (i.e., *degree of crystallinity*) is a critical factor in determining the physical, mechanical, and degradability properties of polymers. Higher crystalline content increases density, hardness, modulus, and solvent resistance, while higher amorphous content imparts softness, elasticity, and ease of processing [17]. Although crystalline ordering in polymers has been extensively studied, amorphous regions—often described by the “bowl of spaghetti” model of randomly coiled chains—are equally important for understanding degradation mechanisms. The interplay between these domains fundamentally governs enzymatic degradation: crystalline areas restrict chain mobility and enzyme access, while amorphous areas provide enhanced molecular mobility and greater accessibility, allowing enzymes to diffuse, bind, and catalyze bond cleavage more efficiently [18, 19]. This structure–property relationship explains why polymers with lower crystallinity generally exhibit faster enzymatic degradation rates, while highly crystalline materials tend to be more persistent in biological environments.

## Phase states and phase transitions in polymers

Polymers exhibit complex thermal behavior characterized by distinct phase transitions that fundamentally govern their physical properties and degradation susceptibility. Three key transition temperatures define polymer behavior: the glass transition temperature (*T*_g_), melting temperature (*T*_m_), and crystallization temperature (*T*_c_). These transitions give rise to four classical thermal states (Table 1), each reflecting different degrees of macromolecular segment mobility [20, 21].

**Table 1:** Overview of polymer states characteristics [10, 20, 23].

Polymer state	Chain mobility	Structural order	Temperature range
Crystalline	Vibrational motions only	Long‐range lamellar order (within crystallites)	Below melting point (*T*_m_) (amorphous fraction may be glassy < _g_ or rubbery > _g_)
Glassy	Localized rotations (frozen chains)	No long‐range order	~ (*T*_g_ – 10 K) to *T*_g_
Rubbery (viscoelastic)	Cooperative segmental motions, relaxation transition	Disordered (amorphous)	Between *T*_g_ and *T*_m_
Melt	Whole‐chain diffusion	None	> *T*_m_

### Four thermal states of polymers

#### Crystalline state

In the crystalline solid state, polymer chains are arranged in long‐range order with a highly organized lamellar structure. This dense packing significantly restricts molecular mobility, resulting in high rigidity, mechanical strength, and thermal stability. The ordered arrangement creates significant barriers to molecular diffusion, including water and enzymes.

#### Glassy state

The glassy state is characterized by disordered chain conformations that are kinetically “frozen” in place. Although localized atomic motions may occur, the system lacks long‐range mobility and exists in a metastable, non‐equilibrium condition with time‐dependent relaxation phenomena, including physical aging and enthalpic recovery.

#### Rubbery (viscoelastic) state

Above the glass transition temperature (*T*_g_), polymers enter the rubbery state, where increased thermal energy enables greater segmental motion. Chains maintain entanglements but possess sufficient free volume to allow elastic deformation under stress [22]. This enhanced mobility significantly facilitates enzyme access to polymer backbones.

#### Melt state

Above the melting temperature (*T*_m_), chain mobility becomes extensive, and the material behaves as a viscous liquid with minimal elasticity, offering theoretically optimal conditions for enzymatic attack—if enzymes can withstand the elevated temperatures [23].

### Glass transition temperature (*T*_g_)

**Fig. 3:**
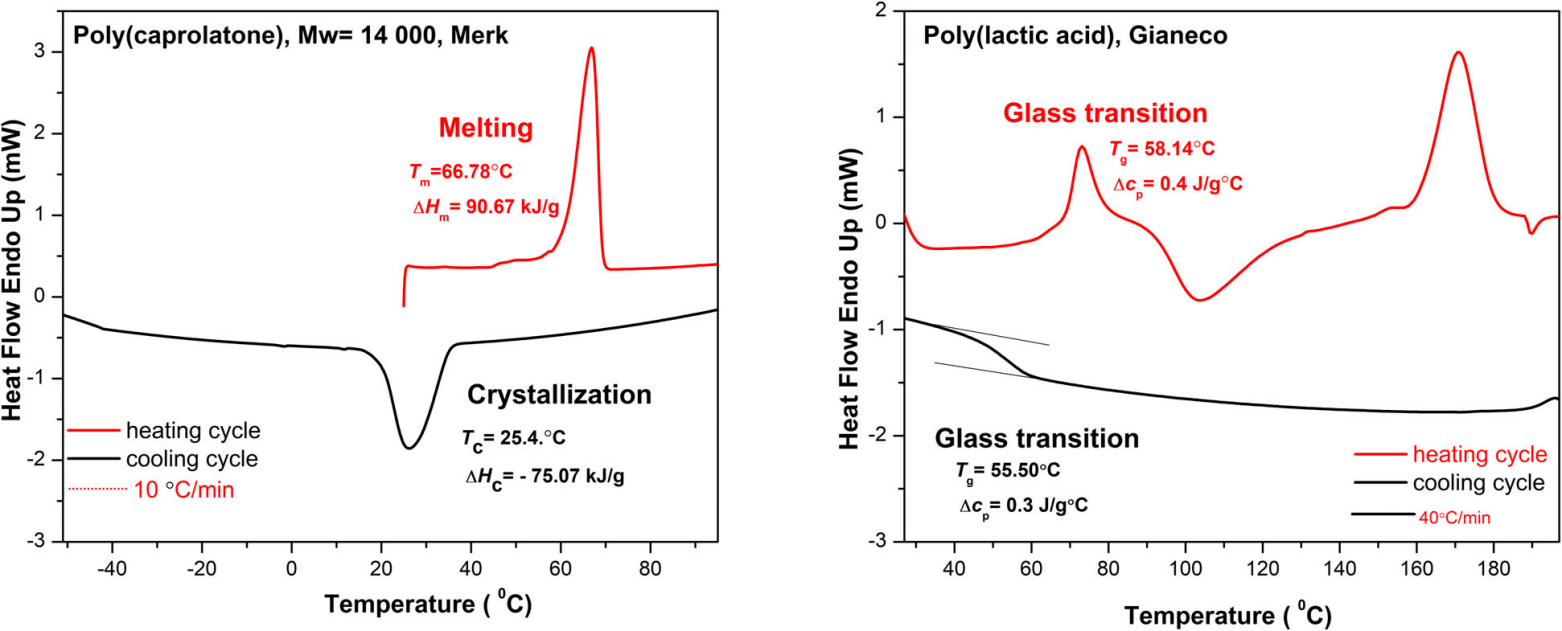
Thermal transitions of semicrystalline polymers measured by Differential Scanning Calorimetry (DSC). Left: First‐order phase transitions (melting and crystallization) of poly(ε‐caprolactone) (PCL), showing melting during heating (endothermic peak corresponding to the absorption of latent heat) and crystallization during cooling (exothermic peak corresponding to the release of latent heat) at a scanning rate of 10 °C·min^−1^. Right: Second‐order glass transition of poly(lactic acid) (PLA) and the associated step change in heat capacity, seen as a shift in the baseline of the heat flow signal, observed during heating and cooling at 40 °C·min^−1^.

The glass transition temperature (*T*_g_) represents a second‐order transition where amorphous polymer chains shift from a rigid, glassy state to a more flexible, rubbery state (Fig. 3). Unlike melting, this transition occurs gradually, without latent heat, and involves progressive changes in molecular mobility rather than an abrupt phase change (PLA as an example in Fig. 3). In semicrystalline polymers, heating beyond *T*_g_ affects only the amorphous regions, while crystalline regions remain structurally intact until the melting temperature (*T*_m_) is reached [10, 20, 23]. As temperature approaches and exceeds *T*_g_, molecular mobility within amorphous regions increases dramatically. This enhanced chain dynamics facilitates enzyme diffusion and access to hydrolysable bonds, making the amorphous regions more susceptible to enzymatic degradation. Operating near or slightly above *T*_g_ can therefore accelerate biodegradation processes, provided enzymes remain structurally stable and catalytically active under these conditions. Key factors affecting *T*_g_ include tacticity, polarity, molecular weight, degree of branching or cross‐linking, and the presence of additives or copolymers [20, 24, 25]. Understanding and controlling *T*_g_ is therefore essential for designing polymers with targeted degradation profiles and predictable environmental behavior.

### Melting temperature (*T*_m_)

Melting represents a first‐order phase transition involving the transformation of crystalline domains into a disordered molten state. Unlike *T*_g_, *T*_m_ applies to the crystalline fraction and involves a sharp change in structure, accompanied by significant enthalpy changes detectable by thermal analysis methods (PCL example in Fig. 3). *T*_m_ is affected by molecular geometry and symmetry, side‐group interactions and polarity, molecular weight, blending or copolymerization and the degree of crystallinity [10, 20, 23]. At temperatures above *T*_m_, polymer chains exhibit maximum mobility, theoretically offering ideal conditions for enzymatic attack [26, 27]. However, most enzymes cannot withstand the thermal stress associated with elevated temperatures at or above typical polymer melting points.

### Crystallization temperature (*T*_c_)

Crystallization is the reverse process of melting and occurs during cooling when polymer chains reorganize from disordered melt into ordered crystalline structures. The crystallization temperature (*T*_c_) depends on chain regularity, flexibility, and intermolecular interactions, and applied cooling rate [10, 20, 23].

Long‐range crystallization is often kinetically hindered due to chain entanglement, resulting in incomplete ordering and the formation of semicrystalline rather than fully crystalline materials.

### Crystallinity and enzymatic degradation

The degree of crystallinity (*x*_c_) serves as a master variable controlling enzymatic degradation behavior. Polymers with lower crystallinity—higher amorphous content—typically degrade more rapidly under enzymatic attack due to greater chain mobility and improved accessibility to target bonds [14, 27, 28].

The effect becomes more evident when degradation occurs near or above *T*_g_ but below *T*_m_, as polymer chains are more mobile while the enzyme operates within its stability range [18, 27, 29]. Conversely, highly crystalline polymers, being mechanically robust and rigid and solvent‐resistant, exhibit slower biodegradation. This resistance arises from the physical inaccessibility of their tightly packed, ordered domains, that severely restrict enzyme diffusion and substrate interaction.

The degree of crystallinity is influenced by multiple structural and processing factors, including chain regularity and symmetry, molecular weight, chain flexibility, intermolecular interactions, polymer composition (random vs. block copolymers), crystallization conditions, cooling rate, thermal history and mechanical treatment [20, 21, 23, 24, 30, 31]. Crystallinity is commonly quantified using complementary analytical techniques such as differential scanning calorimetry (DSC) to analyze thermal transitions and enthalpy‐based crystallinity, density measurements for bulk crystallinity estimation, nuclear magnetic resonance (NMR) for molecular mobility and local order, infrared (IR) spectroscopy for conformational analysis, and X‐ray diffraction (XRD) to determine crystalline vs. amorphous scattering profiles [32, 33].

Given the direct impact of crystallinity on enzymatic and environmental degradation rates, accurate thermal characterization is essential for designing polymers with predictable and controllable degradation behavior (Table 2).

**Table 2:** Brief overview of basic thermal techniques for biopolymer characterization [74, 75].

Thermal method – Principle	Information from method	Parameters
Differential scanning calorimetry (DSC) – measures the difference in heat flow between a sample and a reference material as a function of temperature or time	Overall thermal stability	Temperature degradation, decomposition, oxidation
	First‐order phase transitions such as melting and crystallization, as well as processes like cross‐linking and curing	Melting temperature and enthalpy, *T*_m_, Δ*H*_m_ Crystallization temperature and enthalpy, *T*_c_, Δ*H*_c_ Degree of crystallinity, *x*_c_
	Second‐order phase transitions—glass transition observed during both heating and cooling	Glass transition temperature, *T*_g_ Change in heat capacity Δ*c*_p_
	Purity and quality control	Traces of additives, fillers, solvents, dehydration
Differential thermal analysis (DTA) – measures the difference in temperature between the sample and the reference material	Thermal stability especially at higher temperatures	Temperature decomposition, degradation, denaturation, oxidation
	Detection of melting and crystallization transitions and curing	Melting and crystallization temperature and corresponding enthalpy (*T*_m_, Δ*H*_m_ *T*_c_, Δ*H*_c_ less accurate than DSC) Degree of crystallinity, *x*_c_, can be estimated, though DSC is the preferred method for quantitative determination
	Glass transitions	Glass transition temperature, *T*_g_ Change in heat capacity Δ*c*_p_ (less accurate)
	Quality control	Traces of additives, fillers, solvents, dehydration
Thermogravimetric analysis TGA – measures the mass change of a sample as a function of temperature or time under a controlled atmosphere (commonly nitrogen for inert conditions or air/oxygen for oxidative conditions)	Thermal stability (from room temperature to high temperature)	Thermal degradation/stability, decomposition, oxidation, weight percentage, weight loss (%), *T*_onset_
	Purity information	Moisture content, solvent content and violative temperature
	Composition analysis and estimation of product lifetimes	Residual mass measurements, presence of substances and additives in sample
Dynamic mechanical analysis DMA – measures the viscoelastic properties of a material by applying a small oscillatory deformation (stress or strain) and recording the resulting dimensional/elastic response, usually as a function of temperature, time, or frequency	Thermal stability, relaxation transitions (*α*, *β*, *γ*) and phase transitions (more sensitive than DSC), cross‐linking, curing	Melting, crystallization, glass temperature, relaxation transition or processes in polymers (local motions of polymer groups *T*_*α*_, *T*_*β*_, *T*_*γ*_)
	Mechanical properties and behavior depend on temperature, time and frequency	Storage modulus (*E*′) Loss modulus (*E*″) Damping factor (tang *δ*)
	Miscibility of polymers in polymer blends	Glass temperature *T*_g_ from E′, E″ or tang *δ* data

## Enzymatic degradation mechanisms

### Preferential degradation in amorphous regions

**Fig. 4:**
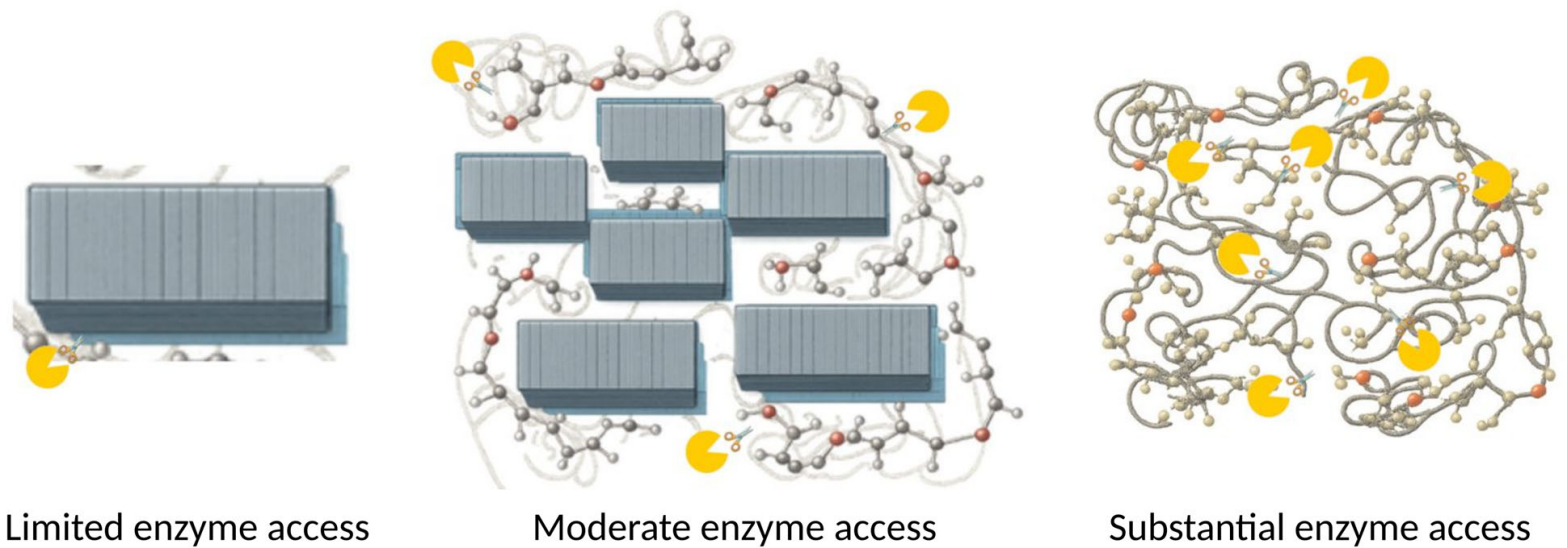
Schematic representation of the enzymatic degradation of polymers with varying crystallinity. In highly crystalline regions, the rigid structure restricts enzyme accessibility and activity, whereas in amorphous regions with greater chain mobility, enzyme access is facilitated, leading to more efficient degradation.

Enzymatic degradation of polymers preferentially occurs in amorphous regions, where increased chain mobility facilitates enzyme access and subsequent chain scission [14, 28, 34]. The enhanced segmental motion and greater free volume in these disordered domains allow enzymes to diffuse more readily, forming productive enzyme‐substrate complexes, catalyzing bond cleavage more efficiently than in densely packed crystalline regions. However, degradation is not exclusively limited to amorphous areas. Crystalline regions can also undergo enzymatic attack [35], particularly at crystallite interfaces, grain boundaries and surface defects where structural imperfections provide enzyme and solvent access points [36]. Additionally, solvent‐mediated swelling can create transient channels within crystalline domains, enabling enzyme penetration and subsequent attack (Fig. 4).

**Fig. 5:**
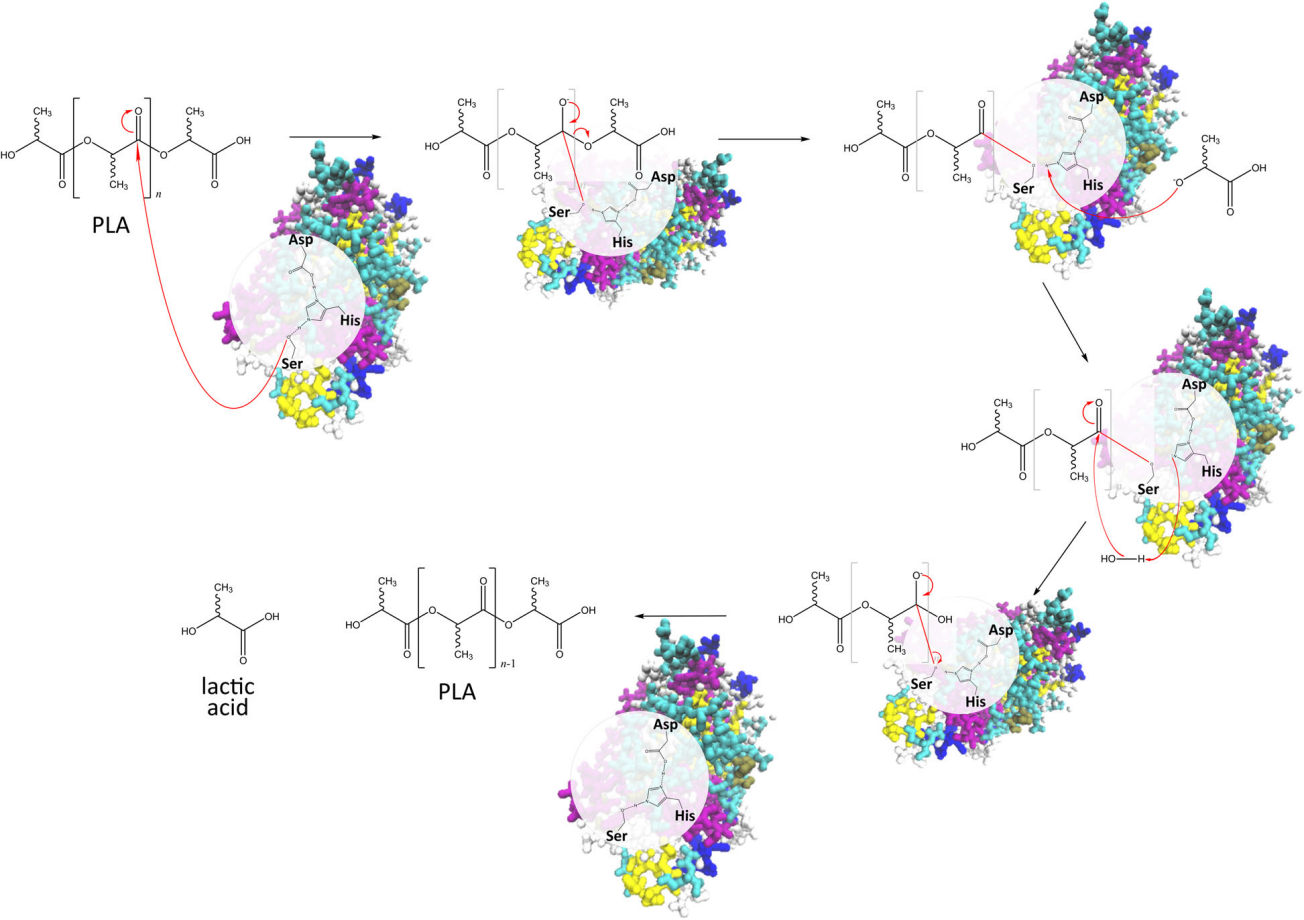
Proposed enzymatic degradation mechanism of poly(lactic acid) (PLA) catalyzed by a polyester hydrolase. The reaction proceeds via a typical serine‐hydrolase mechanism involving the catalytic triad (Ser‐His‐Asp). The enzyme's nucleophilic serine residue attacks the carbonyl carbon of the ester bond in the PLA backbone, forming a tetrahedral intermediate, which subsequently collapses to yield an acyl–enzyme intermediate and a shortened polymer chain. In the following step, a water molecule—activated by histidine—performs a nucleophilic attack on the acyl–enzyme complex, regenerating the free enzyme and releasing the hydrolyzed product. Repeated cycles of these reactions result in progressive chain‐end scission and depolymerization of PLA into lactic acid monomers. The scheme illustrates the catalytic steps and the role of the enzyme's active site in facilitating ester bond cleavage.

Once the enzyme gains access to the amorphous phase of the polymer, degradation proceeds efficiently. Using the depolymerization of poly(lactic acid) (PLA) as an example, enzymatic degradation occurs through a series of hydrolytic cleavage reactions catalyzed at the ester bonds along the polymer backbone. The process involves a nucleophilic attack on the carbonyl carbon, leading to bond cleavage and progressive depolymerization into shorter oligomers and, ultimately, lactic acid monomers (Fig. 5). This schematic representation illustrates the stepwise hydrolysis mechanism and highlights the catalytic role of the enzyme in facilitating PLA chain breakdown.

Recent advances in protein engineering have revealed the importance of conformational selectivity in enzymatic polymer degradation. Notably, engineered PETase variants from *Ideonella sakaiensis*, have been tailored to preferentially bind the trans‐conformation of PET, which is enriched in crystalline domains [37]. This conformational selection proves critical for enzymatic activity: trans‐selective PETase variants exhibited enhanced hydrolysis of crystalline PET due to improved substrate‐enzyme compatibility. Remarkably, even single amino acid substitutions were sufficient to reshape the enzyme's conformational landscape, promoting more productive binding geometries and significantly increasing catalytic efficiency toward trans conformers within ordered crystalline regions in PET [37]. This demonstrates the potential pathway for rational enzyme design to overcome the long‐standing limitations of crystalline polymer degradation.

## Impact of polymer architecture on enzymatic degradation

Polymer architecture – whether a material exists as a homopolymer, random copolymer, or block copolymer (Table 3) – significantly influences enzymatic degradation behavior, and represents a key design parameter for controlling biodegradation rates. Random copolymer incorporation generally increases enzymatic degradability because the irregular monomer sequence disrupts crystal packaging, reduces overall crystallinity and lowers *T*_m_ [18, 38]. For instance, random copolymerization has proven effective for enhancing enzymatic degradation rate of succinate‐based polymers [39], where the incorporation of different diol or dicarboxylic acid comonomers reduces crystallinity and accelerates biodegradation. The irregular chain structure prevents efficient packing of polymer chains, resulting in materials with predominantly amorphous character that are more susceptible to enzymatic attack. Block copolymers exhibit fundamentally different degradation behavior compared to their random counterparts. Block copolymers (e.g., PLA‐*b*‐PCL or PEG‐*b*‐PLA) typically phase‐separate into distinct domains (blocks) with different chemical and physical properties. This phase separation leads to selective enzymatic degradation, where enzymes may preferentially attack one block while leaving the other largely intact. For instance, in PEG‐*co*‐PLA block copolymers, Proteinase K primarily targets PLA segments while PEG regions remain essentially unchanged [40].

**Table 3:** Commonly used commercial biopolymers and block copolymers, along with their molecular structures, degree of crystallinity (*X*_c_), and thermal parameters—melting temperature (*T*_m_), crystallization temperature (*T*_c_), and glass transition temperature (*T*_g_)—representing the thermal window relevant for polymer targeting [18, 49, 50, 51, 52, 53, 74].

Biopolymer	Degree of crystallinity *w*_cx_ (%)	*T*_m_ (°C)	*T*_c_ (°C)	*T*_g_ (°C)
Poly(lactic acid) (PLA) 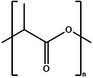	10–40	150–180	80–130	55–65
Poly(glycolic acid) (PGA) 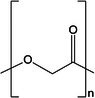	45–55	225–230	140–180	30–40
Poly(L‐lactide‐*co*‐glycolide) (PLGA) (50/50) 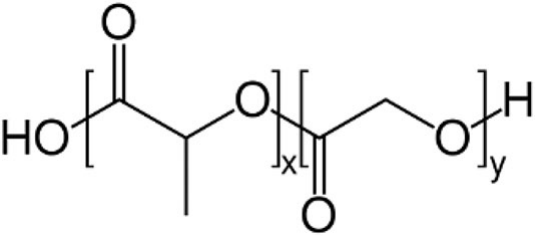	0–10 (Amorphous)	135–155	–	45–55
Poly(ethylene glycol)‐*b*‐poly(lactid acid) PEG*‐b*‐PLA 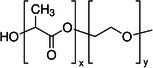	Depends on block length and M_*w*_	40–55 (PEG) 150–170 (PLA)	40–60 (PEG) 80–130 (PLA)	−60 (PEG) 55 (soft blocks of PLA) 40–55 (hard block of PLA)
Poly(hydroxyalkanoate) (PHA) 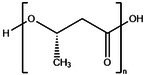	30–70	160–180	60–90	−10 to 5
Poly(hydroxybutyrate) (valerate) (PHB/PHBV) 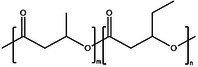	50–70	170–180	80–100	5–10
Poly(caprolactone) (PCL) 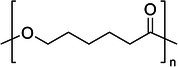	40–60	58–64	30–40	−60
Poly(caprolactone)‐*co*‐Poly(lactic acid) PCL‐*co*‐PLA 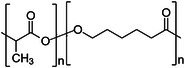	~ 20–30 (PCL) ~ 20 (PLA) in 50/50 copolymer	60–110	~ 30 (PLC)	−40 to 10
Starch thermoplastic (TPS) 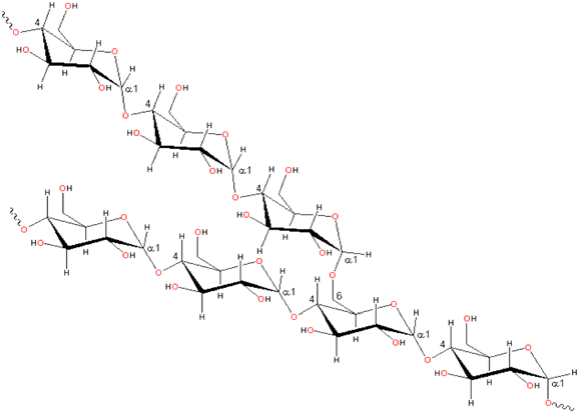	0–30	–	Broad peak	−50 to 20
Cellulose acetate (CA) 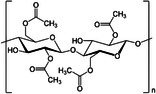	0–20	–	Broad peak	170–190

## Enzymatic degradation in the amorphous regions

A paradigmatic example is the degradation of poly(3‐hydroxybutyrate) (PHB) by extracellular PHB depolymerase from *Alcaligenes faecalis* T1. This enzyme initially hydrolyzes PHB chains in amorphous surface regions before gradually eroding the remaining crystalline domains. Kumagai *et al*., [41] reported that the enzymatic hydrolysis rate of PHB films decreases proportionally with increasing crystallinity, as enzymes must either await spontaneous loosening of amorphous‐crystalline interfaces or actively induce structural disruption before accessing crystalline chains. Importantly, crystalline spherulites' size showed minimal impact on degradation rates, while the fraction of crystallinity proved decisive—low‐crystallinity PHB films degraded substantially faster than high‐crystallinity counterparts under identical conditions.

Poly(lactic acid) (PLA) exhibits similar crystallinity‐dependent degradation, existing in both amorphous (primarily poly(D,L‐lactide)) and semicrystalline variants (poly(L‐lactide)) [34, 42]. In semicrystalline PLA, enzymatic and hydrolytic degradation begins in amorphous domains, where water penetration and enzymes drive rapid molecular weight loss [34, 43]. This preferential attack creates a characteristic biphasic degradation pattern. The first stage is rapid, targeting accessible amorphous regions. As amorphous domains are selectively removed, the material undergoes “crystallinity enrichment” phenomenon—not through new crystal formation, but through selective depletion of the amorphous phase [44, 45]. The remaining crystalline‐rich material exhibits substantially reduced degradation rates, often requiring additional environmental triggers such as elevated temperatures, or prolonged exposure to mechanical stress to disrupt crystalline order for continued enzymatic degradation.

Thermal pretreatment effects [46] demonstrate clear structure–property relationships. Annealing, which increases crystallinity through controlled heating, significantly reduces degradation rates, while rapid cooling (quenching) suppresses crystallization and enhances amorphous content, thereby accelerating degradation under both enzymatic and hydrolytic conditions [1, 47].

## Enzymatic degradation in the molten (or near‐molten) state of low‐melting biopolymers

Certain biopolymers and copolymers possess relatively low melting points including poly(ε‐caprolactone) (PCL, *T*_m_ ≈ 60 °C), [48] poly(lactic acid‐*co*‐glycolic acid) (PLGA, many PLGA compositions are amorphous, showing only glass transition temperatures ≈ 31–60 °C, [47] depending on LA : GA ratio), [49] poly(ethylene glycol)‐*b*‐polylactide (PEG‐*b*‐PLA), [50] poly(ε‐caprolactone‐*co*‐lactide) (PCL‐co‐PLA) [51] making them suitable candidates for studying enzymatic activity under molten‐state conditions (Table 4). The combination of (i) polymers with melting temperatures (*T*_m_) between 45 and 120 °C and (ii) thermophilic or engineered enzymes that maintain activity within this thermal window unlocks a previously inaccessible frontier in biocatalytic recycling: enzymatic depolymerization in the rubbery or fully molten state.

**Table 4:** Thermal window of the target polymers, presented graphically for selected examples [48, 49, 50, 51].

Polymer	*T*_g_ (°C)	*T*_m_ (°C)	Notes	Polymer (typical composition)
PCL (ε‐caprolactone) 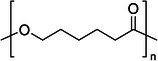	−60	55–62	Fully molten at mild temperatures—ideal test bed	PCL (ε‐caprolactone)
PLGA 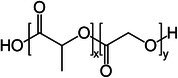	≈ −5 to 15	135–155	*T*g only slightly above ambient; partial melting starts ≈ 130 °C	PLGA (50 : 50)
PEG‐*b*‐PLA 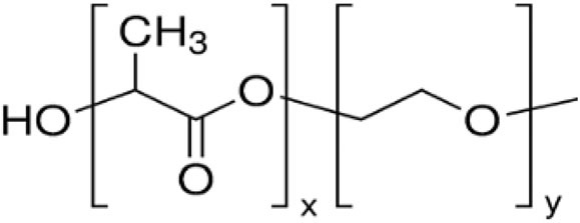	−60 (PEG)/55–60 (PLA)	40–55 (soft block)/150–170 (hard block)	Microphase‐separated: soft PEG melts < 50 °C	PEG‐b‐PLA (MW ≈ 2 kDa/5 kDa)
PCL‐*co*‐PLA 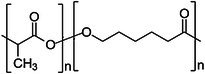	−40 to 10	60–110 (broad)	*T*_m_ depressed relative to neat PLA, enabling “semi‐molten” states at 70–90 °C	PCL‐co‐PLA (random, 50 : 50)

The relatively low melting temperatures of certain aliphatic polyesters, such as PCL [48] and low‐crystallinity PLA, [42] enable polymer chain mobility that approaches small‐molecule liquid behavior at temperatures where many thermostable enzymes retain activity, creating opportunities for molten‐state enzymatic depolymerization that would be impossible with high‐melting‐point polymers like PET without highly specialized enzyme variants.

Several natural and engineered lipases, esterases, and polyester hydrolases exhibit remarkable thermostability and catalytic efficiency near the glass transition temperature of PET (≈ 70 °C). Among them, the thermophilic cutinase variant Leaf‐Branch Compost Cutinase (LCC^ICCG^) shows a melting temperature of ≈ 92 °C and maintains high depolymerization activity at 68 °C, achieving nearly complete (≈ 98%) PET conversion within 24 h while remaining stable throughout the reaction [52]. The hyperthermostable alkaline lipase from *Bacillus sonorensis* 4R exhibited optimal activity at 80 °C and retained a half‐life (*t*_1/2_) of 150 min at this temperature [53] and hyperthermophilic carboxylesterases from extremophilic archaea, such as the *Pyrococcus furiosus* esterase, remain properly folded and catalytically active at 100 °C, exhibiting optimal activity at this temperature and retaining a half‐life of 34 h at 100 °C and 50 min at 126 °C [54, 55]. Beyond naturally robust enzymes, protein engineering has dramatically expanded biocatalyst thermal tolerance. Recent advances in AI‐driven enzyme engineering, ancestral sequence reconstruction (ASR), and structure‐guided stabilization strategies have markedly enhanced the thermostability of PET hydrolases. Using machine learning algorithms, such as MutCompute, researchers have identified mutations that optimize local residue environments and enhance folding robustness, yielding highly stable variants like FAST‐PETase that efficiently depolymerize untreated post‐consumer PET at 50 °C [56]. In parallel, ancestral sequence reconstruction approaches have reconstructed ancient hydrolase ancestors of *Ideonella sakaiensis* PETase, uncovering stabilizing mutations distant from the active site that increase melting temperatures by up to 20 °C while retaining or improving catalytic activity [57]. Complementary strategies, including SpyTag/SpyCatcher cyclization to improve conformational rigidity [58] and rational disulfide bridge incorporation, have further reinforced enzyme stability. Collectively, these methods have produced engineered PETases and cutinases—such as HotPETase, DuraPETase, LCC^ICCG^, and newly evolved PET2‐21 M—with melting temperatures ranging from 80 °C to nearly 99 °C, maintaining high catalytic efficiency under industrially relevant conditions [59, 60, 61, 62].

**Fig. 6:**
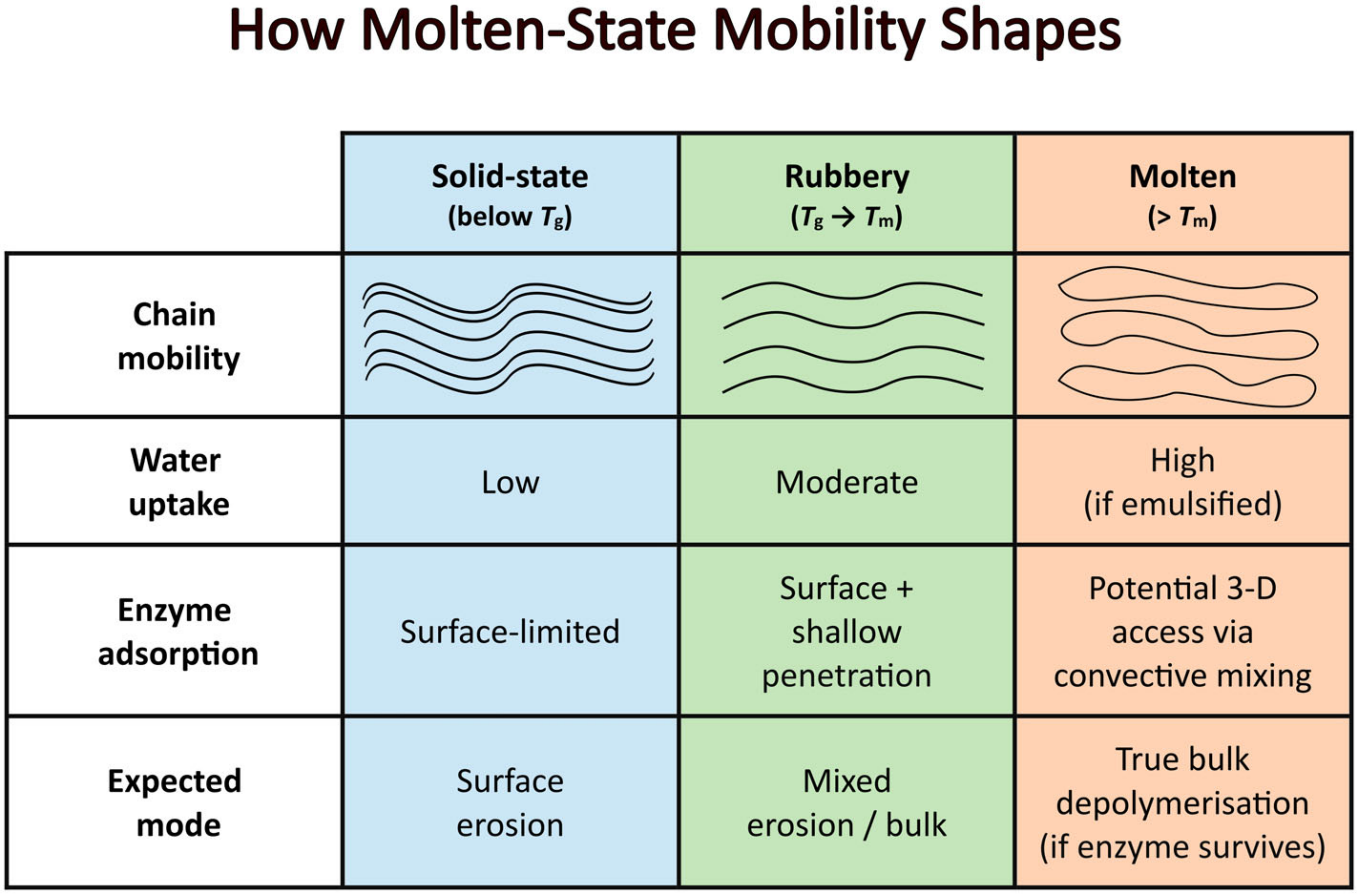
Schematic illustrating how polymer state governs enzymatic depolymerization. As polymers transition from solid (below *T*_g_) to rubbery (*T*_g_ → *T*_m_) and molten (> *T*_m_), chain mobility and water uptake increase, shifting enzyme action from surface‐limited erosion to mixed erosion/bulk degradation—and, when molten and emulsified, enabling 3‐D access via convective mixing for true bulk depolymerization (if the enzyme remains stable).

The physical state of polymers relative to their thermal transitions fundamentally determines enzymatic degradation pathways. Below *T*_g_, in the glassy state, polymer chains remain essentially immobilized with minimal water uptake and enzyme interaction limited to surface adsorption, resulting in surface erosion [63]. In the rubbery regime between *T*_g_ and *T*_m_, enhanced segmental chain motion and improved water absorption enable shallow enzyme penetration, creating a hybrid of surface and limited bulk degradation [63]. In the molten state (above *T*_m_), extensive chain mobility permits free polymer diffusion, significantly enhanced water uptake, particularly in emulsified systems, and potential three‐dimensional enzyme access through convective mixing, enabling true bulk depolymerization provided enzymes maintain structural and catalytic stability at elevated temperatures (Fig. 6). This mechanistic understanding of state‐dependent degradation patterns provides crucial insights for designing next‐generation enzymatic recycling processes that can exploit the enhanced accessibility of molten or near‐molten polymer states while maintaining enzyme functionality through strategic thermostabilization approaches [64].

## Future perspective and conclusion

Most enzymes face significant thermal limitations that prevent direct depolymerization of fully molten polymers, as materials like PLA melt at ≈ 150–170 °C, far exceedingly even the most thermostable enzyme stability ranges. This thermal incompatibility has traditionally restricted enzymatic degradation to solid state conditions, limiting exploitation of enhanced chain mobility in molten states. However, emerging strategies are bridging this gap through innovative protein engineering and materials science approaches.

Thermostable enzyme development represents a critical breakthrough, exemplified by recent advances in PETase and cutinase engineering [65]. The wild‐type IsPETase enzyme [66, 67] has been engineered into a variant known as HotPETase, which features a significantly enhanced melting temperature of approximately 82.5 °C. Additionally, a naturally occurring cutinase from leaf‐branch compost (LCC) has been engineered to exhibit a *T*_m_ of about 94 °C [61]. On the industrial side, Carbios, a French biotechnology company, has progressed toward commercializing enzymatic PET recycling. They are constructing the world's first industrial‐scale PET biorecycling plant in Longlaville, France, which upon completion will have the capacity to process roughly 50 000 tonnes of post‐consumer PET waste per year.

*In‐situ* enzyme incorporation into plastics represents perhaps the most revolutionary approach. DelRe *et al*., [68] presented a breakthrough approach to plastic waste management through nano‐dispersed enzyme embedding, where enzymes are incorporated as nanoparticles (< 2% by weight) directly into semicrystalline polyesters during manufacturing to create self‐degrading materials. The key innovation involves using protective polymer complexes that maintain enzyme stability during high‐temperature processing while enabling activation upon water exposure, leading to processive depolymerization. The results demonstrate up to 98% polymer‐to‐small‐molecule conversion within days for poly(caprolactone) and poly(lactic acid) in standard soil composts and household tap water, with complete elimination of microplastic formation due to the chain‐end‐mediated degradation mechanism.

Building upon this foundation, Guicherd *et al*., [69] developed a commercially viable PLA‐based plastic with embedded hyperthermostable PLA hydrolase, achieving an 80‐fold activity enhancement through structure‐based rational engineering and demonstrating full disintegration under home‐compost conditions within 20–24 weeks using only 0.02% w/w enzyme loading. Their scalable masterbatch‐based melt extrusion process involved incorporating the liquid enzyme formulation into polycaprolactone at 70 °C, then integrating these masterbatch pellets into PLA at 160 °C, producing enzymatic films that maintain mechanical properties compatible with industrial packaging applications while ensuring rapid biodegradation at room temperature.

Computational and machine learning–guided methods are rapidly accelerating enzyme optimization [56, 70], markedly reducing development timelines from years to mere months. For instance, Shao *et al*., [71] introduced EnzyHTP, a high‐throughput computational platform that integrates adaptive resource management with directed evolution strategies to efficiently model and refine enzyme variants *in silico*. Meanwhile, the PRIME language model [72], a temperature‐aware deep learning framework, has demonstrated the ability to predict single‐ and multi‐site mutations that enhance protein stability and activity—over 30% of AI‐suggested mutants outperformed their wild‐type counterparts without experimental mutagenesis. These approaches are part of a broader wave of advances in computational protein design—including Nobel‐recognized efforts [73]—highlighting the transformative power of AI in rapidly designing enzymes with improved thermostability and substrate specificity.

Future research is prioritizing circular economy integration by designing enzymatic recycling pathways that yield high‐value monomers suitable for direct repolymerization. The EU's Horizon Europe program has invested €2.4 billion in enzymatic recycling technologies, while industrial partnerships between companies like Carbios, Eastman, and major brands demonstrate approaching commercial viability.

This review demonstrates that enzyme‐mediated polymer degradation has evolved from fundamental research to practical applications. The integration of advanced enzyme engineering with a sophisticated understanding of polymer thermal behavior is creating opportunities for truly circular systems where biodegradation becomes an integral design feature, promising high‐performing materials that completely biodegrade when their service life concludes—a critical step toward sustainable polymer waste management and environmental impact reduction.

## Conflict of interest

The authors declare no conflict of interest.

## Author contributions

AP and AM contributed equally to the work. Their roles included conceptualization, investigation and literature search, writing of the original draft, review and editing of the manuscript, and preparation of visualizations.
